# Organic Electroluminescent Sensor for Pressure Measurement

**DOI:** 10.3390/s121013899

**Published:** 2012-08-24

**Authors:** Yu Matsuda, Kaori Ueno, Hiroki Yamaguchi, Yasuhiro Egami, Tomohide Niimi

**Affiliations:** 1 Department of Micro-Nano Systems Engineering, Nagoya University, Furo-cho, Chikusa-ku, Nagoya Aichi 4648603, Japan; E-Mails: ueno.kaori@g.mbox.nagoya-u.ac.jp (K.U.); hiroki@nagoya-u.jp (H.Y.); niimi@mech.nagoya-u.ac.jp (T.N.); 2 Department of Mechanical Engineering, Aichi Institute of Technology, 1247 Yachigusa, Yakusa-Cho, Toyota 4700392, Japan; E-Mail: egami@aitech.ac.jp

**Keywords:** optical sensor, pressure-sensitive paint, oxygen quenching, oxygen sensor, organic electroluminescence

## Abstract

We have proposed a novel concept of a pressure sensor called electroluminescent pressure sensor (ELPS) based on oxygen quenching of electroluminescence. The sensor was fabricated as an organic light-emitting device (OLED) with phosphorescent dyes whose phosphorescence can be quenched by oxygen molecules, and with a polymer electrode which permeates oxygen molecules. The sensor was a single-layer OLED with Platinum (II) octaethylporphine (PtOEP) doped into poly(vinylcarbazole) (PVK) as an oxygen sensitive emissive layer and poly(3,4-ethylenedioxythiophene) mixed with poly(styrenesulfonate) (PEDOT:PSS) as an oxygen permeating polymer anode. The pressure sensitivity of the fabricated ELPS sample was equivalent to that of the sensor excited by an illumination light source. Moreover, the pressure sensitivity of the sensor is equivalent to that of conventional pressure-sensitive paint (PSP), which is an optical pressure sensor based on photoluminescence.

## 1. Introduction

Recently, optical measurement techniques based on oxygen quenching of photoluminescence, such as pressure-sensitive paints (PSP) [1–4] and fiber-optic sensors [5], have received much attention due to the fact that the techniques enable us to measure pressure distribution or oxygen concentration with high spatial resolution. A measurement system for these techniques is generally composed of a sensor layer, an illumination light source, a photo-detector, and optics such as optical filters and mirrors [1–5]. Since dye molecules in a sensor layer are photo-excited by an illumination light of a proper wavelength (UV or blue), there are several problems. Firstly, the measurement system needs optical windows or optical fibers to transmit UV light for photo-excitation. This restriction makes the measurement system costly and sometimes complex. Secondly, luminescence from a sensor layer is spatially non-uniform due to the non-uniformity of an illumination light, because the distance from an illumination light source are different at each point on the layer. Thus, a reference signal measured at a known pressure or oxygen concentration condition is required to compensate the non-uniformity of the luminescence by calculating the ratio of a reference signal to a signal obtained at a condition to be measured. However, since a signal is proportional to the intensity of an illumination light, the non-uniformity of the signal to noise ratio (SNR) due to the non-uniformity of an illumination cannot be compensated even with the above-mentioned procedure. This makes the assessment of measurement error or the uncertainty analysis difficult, because it is difficult to measure the distribution of illumination intensity. Thirdly, obstacles in an illumination light path would cast a shadow on a sensor layer, resulting in no emission of luminescence in a shadow region. In general, it is difficult to compensate the effect of a shadow. Lastly, a photo-detector has to be equipped with an optical filter to eliminate the scattering of an illumination light, which is stronger than the luminescence, and this results in reducing the luminescence and SNR detected by a photo-detector.

Recently, Iijima and Sakaue [6] proposed a new illumination method for PSP to improve the non-uniformity of an illumination. They applied PSP to the top surface of an inorganic EL device; thus, PSP is illuminated from underside by the inorganic EL device. However, the emission spectra of PSP overlap with that of the inorganic EL, which resulted in low pressure sensitivity and SNR.

We propose a novel concept of a pressure sensor based on not photoluminescence but electroluminescence. In other words, an organic light-emitting device (OLED) [7–11] which *itself* works as a pressure sensor has been proposed. The proposed sensor is called the electroluminescent pressure sensor (ELPS) in this paper. An illumination light source is no longer necessary and ELPS is free from the problems written above due to the usage of an illumination light.

## 2. Materials and Methods

### 2.1. Photophysical Principles of ELPS

ELPS emits electroluminescence based on OLED process [12,13], and works as a pressure sensor in the same principle with PSP except for the usage of an illumination light. In this study, we adopted a dopant material, whose luminescence can be quenched by oxygen, incorporated into a host material. Since the luminescence can be quenched by oxygen molecules, the luminescent intensity decreases as the oxygen concentration increases in the emissive layer. The oxygen concentration in the emissive layer is proportional to the partial pressure of oxygen or air pressure, p, according to the Henry's law and the Dalton's law [1,2]. The relation between luminescent intensity and the oxygen concentration or pressure is written as the Stern–Volmer equation

(1) $$\frac{I_{\text{ref}}}{I}=A_{c}+B_{c}\frac{\left[{\text{O}}_{2}\right]}{{\left[{\text{O}}_{2}\right]}_{\text{ref}}}=A_{p}+B_{p}\frac{p}{p_{\text{ref}}},$$

where *I* is the luminescent intensity and the subscript “ref” describes a known (reference) condition. [O_2_] is oxygen concentration and the coefficients A and B are the Stern–Volmer coefficients, which are determined by a calibration test. The calibration data are fitted by Equation 1 with the constraint condition of *A_c_* + *B_c_* = 1 at [O_2_]/[O_2_]_ref_ = 1, *I*_ref_/*I* = 1 (or *A_p_* + *B_p_* = 1 at *p*/*p*_ref_ = 1, *I*_ref_/*I* = 1), because the data are normalized by the values at the reference condition, [O_2_]_ref_ (or *p*_ref_) and *I*_ref_. The Stern–Volmer equation shows the dependence of the luminescent intensity on oxygen concentration or pressure; thus, the slope of Equation 1,*B_c_* or *B_p_*, shows the oxygen or pressure sensitivity. The oxygen concentration sensitivity corresponds to the pressure sensitivity in this formula [4]. Therefore, this sensor works as both oxygen and pressure sensor. In this study, the coefficient *B_p_* was determined; that is, the pressure sensitivity of ELPS was investigated.

### 2.2. Fabrication

**Figure 1.**
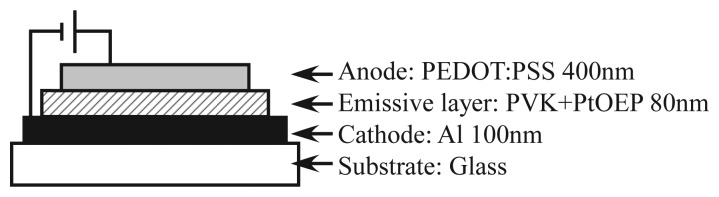
Configuration of ELPS.

Figure 1 shows the configuration of ELPS. A single-layer OLED configuration was adopted to simplify the fabrication procedure. Platinum (II) octaethylporphine (PtOEP), which is used as both oxygen sensor [2] and OLED [14], was adopted as an oxygen probe. The pressure or oxygen sensitivity of PtOEP was investigated in detail by Amao and his coworkers [15,16]. PtOEP was doped into the host material poly(vinylcarbazole) (PVK) at the ratio of 1:9 w/w PtOEP/PVK. An aluminum cathode was vapor-deposited on a glass plate. To achieve high oxygen permeation, a polymer anode, poly(3,4-ethylenedioxythiophene) mixed with poly(styrenesulfonate) (PEDOT:PSS) [17–19], was employed. Since the anode is transparent, the luminescence can be detected through the anode. In general applications of OLED such as display or lighting, the permeation of oxygen molecules has to be prevented because the permeation of oxygen molecules will induce oxidation of electrodes. In the viewpoint of the pressure sensor, the permeation of oxygen molecules is essential for oxygen quenching, and the sensor is restricted to a short lifetime. However, since general optical sensors like PSP are disposable, the long lifetime of the sensor is not required and a simple fabrication procedure is important.

The fabrication process was as follows. Firstly, the solution of PtOEP (Frontier Scientific) and PVK (Tokyo Chemical Industry) in toluene (10 mg/mL) was poured on the cathode deposited on the glass plate, which was preheated at 60 °C. Then, the glass plate was spun at 800 rpm to make a thin emissive layer. The emissive layer was dried for 5 min by putting on a hot plate kept at 130 °C. Since PtOEP and PVK are hydrophobic materials, the surface of the emissive layer was treated by an O_2_ plasma asher (J-Science lab, JPA300) to change the surface to hydrophilic. The PEDOT:PSS water solution (Orgacon, HBS5) with the addition of 8 vol% glycerol [20,21] was spin-coated at 400 rpm for 2 min and 500 rpm for 1 min. Following the procedure proposed by Snaith *et al.* [22], the anode film was annealed at 80 °C for 1 hour followed by 120 °C for 30 min. The thickness of the each layer presented in Figure 1 was measured by Surfcorder ET200 (Kosaka Laboratory). The emission area of the fabricated ELPS was 7 mm × 9 mm. The ELPS sample emits the light of peak wavelength of 650 nm, which emits from PtOEP, and does not emit the blue light from a host material of PVK. That is, all emission from ELPS is due to PtOEP, which works as an oxygen probe.

### 2.3. Experimental Setup

**Figure 2.**
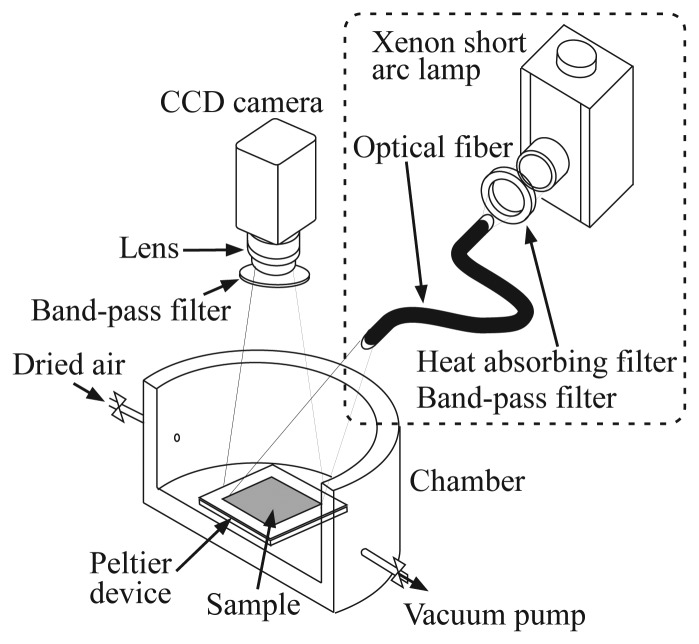
Schematic image of experimental setup. The illumination light source inside the dashed box is only used for the photoluminescence experiment.

Figure 2 shows the experimental setup for the investigation of the pressure sensitivity of the ELPS sample. The pressure sensitivities were examined in a chamber, where the pressure was controlled from 3 to 101 kPa by dried air and a vacuum pump (ULVAC, DAP-6D). The ELPS sample was placed on a stage with a peltier device in the chamber and the temperature of the sample was kept at 20.0 ± 0.1 °C to eliminate the effect of thermal quenching induced by Joule heating. A luminescence emitted from the sample was detected by a 12 bit CCD camera (LaVision, Imager Compact) with a band-pass filter (Asahi-spectra, 630 ± 60 nm) to eliminate an illumination light used for photo-excitation. Although the band-pass filter equipped on a CCD camera is not required for ELPS measurement, we used it to compare the pressure sensitivity of the ELPS sample with that excited by an illumination light under the same condition. When we investigated the pressure sensitivity of the photo-excited sample, the sample was illuminated by a xenon short arc lamp (Ushio, UI-502Q) with a heat absorbing filter (Sigma Koki) and a band-pass filter (Sigma Koki, 400 ± 20 nm). The intensity data of 55 × 85 pixel (about whole of the emission area) were averaged to reduce the shot noise.

## 3. Results and Discussion

**Figure 3.**
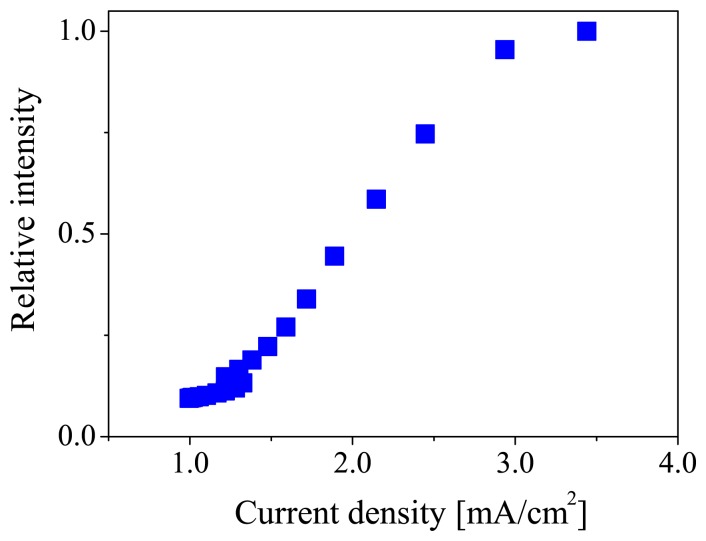
Luminescent intensity against current density for the ELPS sample.

Figure 3 shows the current density-luminescent intensity characteristic of the ELPS sample. The vertical axis is the luminescent intensity normalized by the maximum luminescent intensity at 17.5 V. This characteristic is similar to the device reported by Burroughes *et al.* [24]. The luminescent intensity monotonically increased with increasing current density in the range from 1.5 to 3 mA/cm^2^. In this range, the ELPS sample can stably emit electroluminescence. No degradation of OLED performance was observed during the measurement.

**Figure 4.**
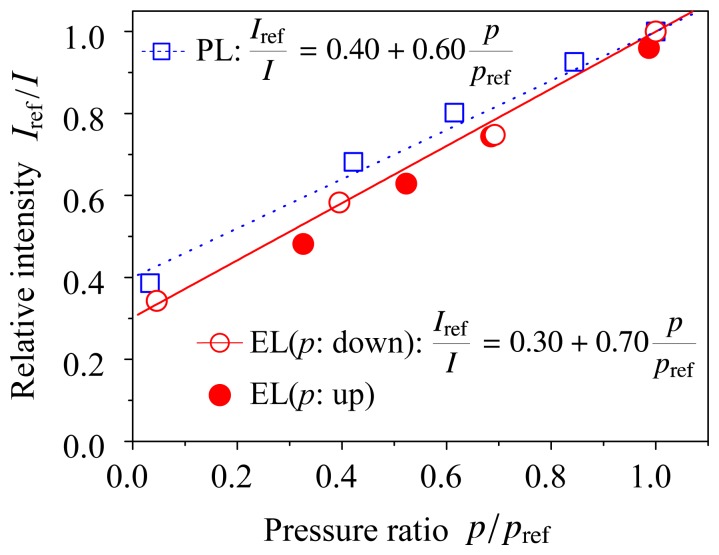
Stern–Volmer plots of electroluminescence (EL) and photoluminescence (PL), where the reference pressure *p*_ref_ = 101 kPa. The data shown in open circle were obtained first with decreasing pressure, and the data shown in closed circle were obtained with increasing pressure.

Figure 4 shows the Stern–Volmer plots for the ELPS sample both electroluminescence (EL) and photoluminescence (PL) experiments, where the reference pressure *p*_ref_ = 101 kPa and the *I*_ref_ is the luminescent intensity at the reference pressure. The voltage and current were kept at 13 V and 1.3 mA (2 mA/cm^2^), respectively, because the luminescent intensity under a constant voltage and current condition depends only on pressure or oxygen concentration. We first obtained the EL data with decreasing pressure in the chamber (open circle), and then obtained the data with increasing pressure (closed circle). It was shown that the hysteresis of the EL intensity was small and the ELPS did not degrade during the measurement. The solid and dotted lines in Figure 4 are the fitted line for the experimental data of electroluminescence and photoluminescence, respectively. The experimental data were well fitted by Equation 1 and the inverse of luminescent intensity, 1/*I*, linearly depends on pressure *p*. This is a preferable characteristic for pressure sensor [2]. Pressure sensitivities of the ELPS sample were *B_p_* = 0.70 ± 0.06 for electroluminescence and *B_p_* = 0.60 ± 0.07 for photoluminescence, respectively. The errors in the pressure sensitivities were calculated as a fitting error (95% confidence interval) of the experimental data with Equation 1. The pressure sensitivity of EL was slightly higher than that of PL. A possible reason of this difference in pressure sensitivities is photo-degradation of dye and polymers. However, it is considered that the pressure sensitivity of the ELPS sample is independent of excitation method, when the confidence intervals are considered. The pressure sensitivity of EL is similar to that of conventional PSPs reported by Liu and Sullivan[2]. This result shows that ELPS works as a pressure sensor without an illumination light source.

## 4. Conclusions

We proposed a novel concept of a pressure sensor called electroluminescent pressure sensor (ELPS) based on oxygen quenching of electroluminescence. In this study, we investigated the pressure sensitivities of the ELPS sample using both electroluminescence and photoluminescence, showing that the pressure sensitivity of ELPS is equivalent to that of conventional PSPs. Moreover, it was clarified that the hysteresis of the EL intensity was small and the ELPS did not degrade during the measurement. The oxygen concentration sensitivity corresponds to the pressure sensitivity, thus the proposed sensor will also work as an oxygen sensor.
